# Information Transfer via Gonadotropin-Releasing Hormone Receptors to ERK and NFAT: Sensing GnRH and Sensing Dynamics

**DOI:** 10.1210/js.2016-1096

**Published:** 2017-02-27

**Authors:** Kathryn L. Garner, Margaritis Voliotis, Hussah Alobaid, Rebecca M. Perrett, Thanh Pham, Krasimira Tsaneva-Atanasova, Craig A. McArdle

**Affiliations:** 1 Laboratories for Integrative Neuroscience and Endocrinology, School of Clinical Sciences, University of Bristol, Bristol BS1 3NY, United Kingdom;; 2 EPSRC Centre for Predictive Modelling in Healthcare, and; 3 Department of Mathematics, College of Engineering, Mathematics and Physical Sciences, University of Exeter, Exeter EX4 4QF, United Kingdom; and; 4 Texas A&M University Corpus Christi, Corpus Christi, Texas 78412

**Keywords:** extracellular signal-regulated kinase, nuclear factor of activated T cells, G protein coupled receptor, gonadotropin-releasing hormone, mitogen-activated protein kinase, mathematical modeling

## Abstract

Information theoretic approaches can be used to quantify information transfer via cell signaling networks. In this study, we do so for gonadotropin-releasing hormone (GnRH) activation of extracellular signal-regulated kinase (ERK) and nuclear factor of activated T cells (NFAT) in large numbers of individual fixed L*β*T2 and HeLa cells. Information transfer, measured by mutual information between GnRH and ERK or NFAT, was <1 bit (despite 3-bit system inputs). It was increased by sensing both ERK and NFAT, but the increase was <50%. In live cells, information transfer via GnRH receptors to NFAT was also <1 bit and was increased by consideration of response trajectory, but the increase was <10%. GnRH secretion is pulsatile, so we explored information gained by sensing a second pulse, developing a model of GnRH signaling to NFAT with variability introduced by allowing effectors to fluctuate. Simulations revealed that when cell–cell variability reflects rapidly fluctuating effector levels, additional information is gained by sensing two GnRH pulses, but where it is due to slowly fluctuating effectors, responses in one pulse are predictive of those in another, so little information is gained from sensing both. Wet laboratory experiments revealed that the latter scenario holds true for GnRH signaling; within the timescale of our experiments (1 to 2 hours), cell–cell variability in the NFAT pathway remains relatively constant, so trajectories are reproducible from pulse to pulse. Accordingly, joint sensing, sensing of response trajectories, and sensing of repeated pulses can all increase information transfer via GnRH receptors, but in each case the increase is small.

## Teaser

Information transfer via GnRHR is increased by joint sensing of ERK and NFAT and by sensing trajectories, but the increases are small, as most information is lost through these noisy signaling pathways.

Single-cell measures of cell signaling pathways and proteins reveal marked cell–cell variation, but relatively little is known about the biological relevance of this heterogeneity. It is, in fact, inevitable because the processes underlying signaling are stochastic, and it is thought to be crucial for the behavior of cell populations [1] where each individual cell has to sense the environment and make appropriate decisions (to express or suppress given genes, for example). Information theoretic approaches are increasingly being applied to cell biology, where cell signaling systems can be treated as noisy communication channels and statistical measures of information transfer that take into account cell–cell variation can be calculated [1–8]. In this study, information is defined as the uncertainty about the environment that is reduced by signaling and is measured as the mutual information (MI) between two stochastic variables describing the signal and the response [1]. For cell signaling pathways, these variables could be the concentration of stimulus in the environment and the activity of an effector. In this case, MI quantifies the quality of the inference of the signal from the response, providing a statistical measure of information transfer through the pathway.

This information theoretic approach takes variability into account rather than just considering the average response, can be applied to any cell signaling system in which signal and response are known, and can provide significant additional insight into cell signaling. Its merit can be illustrated by consideration of a signaling network with multiple possible routes of information transfer, as it would theoretically be possible to measure the amount of information passing from any given receptor to any given effector. The network may well contain numerous potential drug targets, but the pathways and effectors providing the cell with most information about the environment would presumably make the most attractive targets for therapeutic manipulation. Alternatively, for a simple multitiered signal-transduction pathway it is often assumed that signal amplification occurs through the cascade. However, information theory tells us that information about the signal cannot actually increase from one tier to the next, so any increase in numbers of activated molecules must be associated with increased variability. In fact, there is almost inevitably loss (and never gain) of information through such cascades. Indeed, marked loss of information has already been documented for several signaling pathways, including for tumor necrosis factor signaling to nuclear factor *κ*B [2], for nerve growth factor and pituitary adenylyl cyclase–activating polypeptide signaling to cyclic adenosine 5′-monophosphate response element binding protein, c-FOS, and Egr1 [7], and for epidermal growth factor signaling to extracellular signal-regulated kinase (ERK) [4]. In each of these cases, ~3 bits of information were available (*i.e.*, ~2^3^ states of the environment were considered) but <1 bit of information was typically transferred. This raises the question of how cells could mitigate this loss, and emphasis has been placed on negative feedback loops that could reduce information transfer by reducing the dynamic range of the output, or increase it by reducing cell–cell variability or by preventing basal stimulation due to constitutive protein activity [2, 4, 9].

In this study, we explore information transfer via gonadotropin-releasing hormone receptors (GnRHRs) to ERK. GnRHRs are G_q/11_-coupled G protein–coupled receptors in the pituitary that mediate central control of reproduction [10]. When activated by the neuropeptide GnRH they cause a phospholipase C (PLC)–mediated increase in the cytoplasmic Ca^2+^ concentration that drives exocytotic gonadotropin secretion. This Ca^2+^ elevation also has marked effects on transcription, in part mediated by the Ca^2+^/calmodulin-mediated activation of nuclear factor of activated T cells (NFAT) [10, 11]. GnRHR-mediated PLC activation also activates protein kinase C isozymes and causes a (largely) protein kinase C–mediated activation of ERK and of ERK-driven transcriptional responses [10, 12–18]. Where single-cell measures are available they typically reveal marked cell–cell variability, even in clonal cell models. Thus, GnRH effects on cytoplasmic Ca^2+^ concentration, gonadotropin secretion, effector activation, and gene expression all show pronounced heterogeneity in normal pituitary cells as well as in gonadotrope lineage cell lines and in heterologous receptor expression systems [9, 11, 17–24]. Using a high-content imaging system to obtain signaling measures from large numbers of individual cells and MI to quantify GnRH sensing, we recently showed that there was a marked loss of information through signaling, in that for most experiments there were 3 bits of information available but information transfer was always <1 bit. In this study, we address a number of possible reasons for this low level of information transfer. We test the relevance of cellular context and effector choice by quantifying MI between GnRH and double phosphorylated ERK (ppERK) or NFAT using upstream and transcriptional readouts in HeLa cells and in L*β*T2 (gonadotrope lineage) cells. We also address the possibility that additional information is gained by joint sensing of both of these pathways or by sensing response trajectories over time, using wet laboratory data and by developing a hybrid mechanistic/probabilistic model of GnRH signaling to NFAT. Our key findings are that information transfer can indeed be increased by joint sensing (of ERK and NFAT) and by sensing NFAT over time, but in each case the additional information gained is relatively small. Indeed, MI values were <1 bit under all conditions considered, suggesting that most information about GnRH concentration is actually lost by GnRH-responsive cells such that these individual cells cannot unambiguously distinguish between even two states of the environment.

## 1. Materials and Methods

### A. Cell Culture and Transfection

The murine gonadotrope-derived L*β*T2 cell line was provided by Prof. P.L. Mellon (University of California San Diego, San Diego, CA). The cells were routinely cultured in Dulbecco’s modified Eagle’s medium (DMEM), 10% heat-inactivated fetal calf serum (FCS) (Gibco), 100 U/mL penicillin, and 0.1 mg/mL streptomycin (Sigma-Aldrich) in Matrigel basement membrane matrix (BD Biosciences)–coated tissue culture flasks, and were plated (10 × 10^3^ cells per well) in Costar black-walled 96-well plates (Corning) for imaging experiments. For most experiments the plated cells were also transduced with recombinant adenovirus (Ad) for expression of an NFAT1c–emerald green fluorescent protein (EFP) translocation reporter [11]. Approximately 16 hours after plating they were incubated 4 to 6 hours in DMEM/2% FCS with Ad NFAT1c-EFP. The medium was then replaced with DMEM/0.1% FCS and the cells were incubated for a further 16 hours before stimulation as described [18]. For some experiments imaging readouts for pathway-specific transcriptional responses were obtained by transducing cells with Ad for expression of an Egr1 promoter driving expression of zsGREEN (Ad Egr1-zsGREEN) or with Ad for expression of an NFAT response element (RE) driving expression of asRED (Ad NFAT-RE asRED) as described [25]. As an alternative cellular model, HeLa cells (from the European Collection of Authenticated Cell Cultures) were used. They were cultured, plated, and transduced as described [25, 26] and, because HeLa cells do not express endogenous GnRHR, they were also transduced with Ad GnRHR as described [25–28]. In this model GnRHR number is dependent on Ad titer, and Ad GnRHR was used at 1 to 2 plaque-forming units/nL to provide receptor expression at ~50,000 sites per cell [9], which is within the range of 20,000 to 75,000 sites per cell estimated for endogenous GnRHR in gonadotrope lineage cells and primary cultures [9, 29]. All other Ads were used at 1 to 10 plaque-forming units/nL, and stimulation details are given in the figure legends.

### B. Image Acquisition and Analysis

For the first experiments L*β*T2 cells or Ad GnRHR-transduced HeLa cells were stimulated for varied periods with varied concentrations of GnRH before being fixed and stained with 4′,6′-diamidino-2-phenylindole (DAPI) for visualization of nuclei and with anti-ppERK antibody (Sigma-Aldrich, catalog no. M9692; RRID:AB_260729) followed by Alexa Fluor goat anti-mouse fluorescent secondary antibody (Molecular Probes) [25–28]. Digital images were then acquired with an InCell Analyzer 1000 high-content imaging platform (GE Healthcare) using a 10× objective and filters for DAPI (blue channel) and Alexa Fluor 488 (green channel; Thermo Fisher Scientific, catalog no. A-11029; RRID_2534088) or Alexa Fluor 546 (red channel; Thermo Fisher Scientific, catalog no. A-11030; RRID:AB_2534089). For some experiments, NFAT1c-EFP, zsGREEN, or asRED were also visualized (using green and red channel filters as appropriate). Automated image analysis was as described [27] determining whole-cell or nuclear fluorophore intensities in arbitrary fluorescence units. For the NFAT1c-EFP translocation assay, background-subtracted cytoplasmic and nuclear fluorescence intensities were used to calculate the fraction of NFAT1c-EFP in the nucleus [NFAT–nuclear fraction (NF)]. Replicate treatments in two to four wells of cultured cells were pooled to produce population-averaged responses that were pooled from multiple experiments with ppERK [25–28].

For live cell imaging experiments, HeLa cells cultured, plated, and transduced with Ad GnRHR and Ad NFAT1c-EFP as shown earlier were transferred to live cell imaging buffer [20 mM HEPES (pH 7.4), 137 mM NaCl, 5 mM KCl, 2 mM MgCl_2_, 1.8 mM CaCl_2_, 5.6 mM glucose, 1 mg/mL bovine serum albumin, 0.5 mM NaH_2_PO_4_, 1 mM NaHCO_3_, 0.03 mM phenol red] and stained with Hoechst 33342 dye (Molecular Probes) for 30-minute equilibration, serum starvation, and nucleus staining. The cells were imaged at 37°C both before and during stimulation with GnRH (0, 10^−11^, 10^−9^, or 10^−7^ M). For the first live cell imaging experiment, the cells received a single 60-minute pulse of GnRH, but for the second experiment there were two pulses: a 15-minute pulse terminated by extensive washing (four changes of medium) followed by an interval of 135 minutes, and a subsequent 60-minute pulse of GnRH (at the same concentration as had been used for the first pulse). Digital images were acquired (at the time points indicated in the figures) and individual cells were tracked over time (see later) so that NFAT-NF could be plotted against time for each individual tracked cell. These individual cell time courses were inspected for removal of cells in which tracking had failed and outliers with time 0 NFAT-NF values of <0.4 or >0.55 (this removed <10% of the cells from three repeated experiments). The figures show representative individual cell responses, as well as population-averaged responses for all tracked cells.

### C. Data Analysis

For the initial fixed cell experiments, we constructed full concentration response curves (*i.e.*, control and 10^−12^ to 10^−6^ M GnRH) at multiple time points and collected images for four to nine fields of view per well. This yielded measures of nuclear ppERK or NFAT-NF for >10,000 individual cells (for each treatment in each experiment). For some experiments GnRH potency was estimated by curve fitting of population average responses using GraphPad Prism [log (agonist) vs response, four parameter fit using Prism 6 for Windows, version 6.05]. Additionally, for most experiments individual cell measures from complete concentration–response curves were used to calculate MI between stimulus concentration and the experimental measure at each time point. MI was estimated using the following formula:

*I*(*Z*;*S*) = *H*(*Z*) − *H*(*Z*|*S*),

where *I* is the MI between a signal (*S*) and a response (*Z*), *H*(*Z*) is the unconditional entropy of the response, and *H*(*Z*|*S*) is the conditional entropy [4]. To estimate these entropy terms we used the Bayesian method proposed by Nemenman *et al.* [30], which also provides error bars for these estimates. As the method is designed for discrete data, we discretized ERK and NFAT cell measures by binning them into 30 equally sized bins.

For the second series of fixed cell experiments, data were collected (as shown earlier) for nuclear ppERK and NFAT-NF in the same cells, or for Egr1-driven zsGREEN and NFAT-RE–driven asRED, again in the same cells. This enabled us to calculate not only the MI between GnRH and each individual experimental measure, but also the joint MI between GnRH and the paired outputs (ppERK and NFAT-RE or asRED and zsGREEN) as previously shown, but with response (*Z*) now interpreted as a two-dimensional vector.

For the live cell imaging experiments, cells were initially segmented from the DAPI images using InCell Analyzer software. Individual cells were then tracked by matching the geometric centers of the nuclei between successive images in the time stack. Cells were paired with probability that depended exponentially on their Euclidean distance, and Markov chain Monte Carlo was used to find the most likely matching configuration for each pair of images.

For the single-pulse live cell imaging experiments, MI values were then calculated between GnRH and the NFAT-NF translocation response at individual time points. Additionally, MI values were calculated between GnRH and the integral of the NFAT-NF translocation response (during the 60-minute stimulation period) or using three time points to take response trajectory into consideration. For the dual-pulse live cell imaging experiments, the responses (*Z*_1_ and *Z*_2_) to the first and second pulse were measured as the maximum NFAT-NF value during each pulse. Information *I*(*Z*_1_;GnRH) was calculated, and the additional information from the response to the second pulse was calculated using the following formula:

*I*(*Z*_1_;*S*|*Z*_1_) = *I*(*Z*_1_;GnRH) − *I*(*Z*_1_;*Z*_2_) + *I*(*Z*_1_;*Z*_2_|GnRH),

where *I*(*Z*_1_;*Z*_2_) is the MI between the individual cell responses in pulses 1 and 2, and *I*(*Z*_1_;*Z*_2_|GnRH) is the MI between the individual cell responses in pulses 1 and 2 conditioned on the concentration of GnRH.

All of the analyses were performed in MatLab (MathWorks).

### D. Simulations of the Hybrid (Deterministic/Stochastic) Model

We used a deterministic model of GnRH signaling that was adapted from an earlier version [31] by removal of the ERK signaling pathway and transcription regulation steps and by altering the NFAT translocation parameters to better fit the response dynamics shown for wet laboratory data from the two pulse experiments. We introduced stochastic dynamics for two effectors in the model, GnRHR (the first step in the pathway) and calmodulin (which equates to parameter *M* in Ref. [31]), by allowing the corresponding parameters (describing the total amount of these effectors) to fluctuate over time according to an exponentiated Ornstein–Uhlenbeck process: accordingly, stationary mean was set to the value of the corresponding parameter in the deterministic model, stationary variance was set such that the response variability matches the one observed experimentally, and fluctuation lifetime (FL) was set to vary between 10 minutes (unstable effector) and 10,000 minutes (stable effector). We used the hybrid model to simulate responses to two pulses of 0, 10^−11^, 10^−9^, and 10^−7^ M GnRH. The first pulse was for 15 minutes, and this was followed by a 135-minute interval and then a second pulse (of 60 minutes). As with the wet laboratory data, we measured the responses to the first and second pulse (*Z*_1_ and *Z*_2_) as the maximum NFAT-NF value during each pulse. We ran simulations for 1000 cells at each GnRH concentration/FL combination, and used the simulated responses to calculate *I*(*Z*_1_;GnRH) and the additional information from *Z*_2_ as detailed earlier.

## 2. Results

### A. Statistical Measurement of Information Transfer via GnRHR to ERK and NFAT

**Figure 1.**
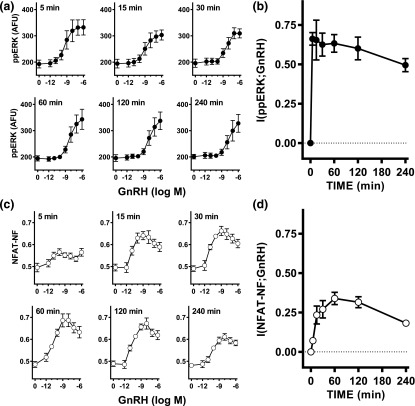
Quantifying information transfer via GnRHR to ERK and NFAT in L*β*T2 cells. (a) L*β*T2 cells in 96-well plates were continuously stimulated for varied periods with 0 or 10^−12^ to 10^−6^ M GnRH as indicated and then fixed and stained (DAPI and ppERK) before image capture and analysis. The data shown are population-averaged nuclear ppERK measures in arbitrary fluorescence units (AFU) and are means ± standard error of the mean from three separate experiments, each with triplicate wells (n = 3). Background values (without fluorophore) were 120 to 150 AFU and were not subtracted. Log EC_50_ values were −9.03 ± 0.39, −8.07 ± 0.16, −7.95 ± 0.19, −8.01 ± 0.53, −7.76 ± 0.60, and −7.34 ± 0.74 at 5, 15, 30, 60, 120, and 240 minutes, respectively. One-way analysis of variance revealed time to be a significant variable (*P* < 0.05), and *post hoc* Bonferroni tests (comparing to the 5-minute data) revealed a significant difference at 240 minutes (*P* < 0.05) but not at any other time point. (b) The single-cell ppERK measures from the full concentration response curves in (a) were used to calculate the MI between GnRH concentration and ppERK at each time point, and these I(ppERK;GnRH) values (in bits) are plotted against time. (c) Ad NFAT-EFP–transduced L*β*T2 cells in 96-well plates were stimulated for varied periods with 0 or 10^−12^ to 10^−6^ M GnRH as indicated and then fixed and stained (DAPI) before image capture and analysis. NFAT-EFP fluorescence intensity was determined for the nucleus and cytoplasm and used (after subtraction of background values, which were ~200 AFU) to calculate the proportion of NFAT-EFP in the nucleus. The data shown are population-averaged measures of this NFAT-NF and are means ± standard error of the mean from three separate experiments, each with triplicate wells (n = 3). (d) The single-cell NFAT-NF measures from the full concentration response curves in (c) were used to calculate the MI between GnRH concentration and NFAT-NF and these I(NFAT-NF;GnRH) values (in bits) are plotted against time. The data in (a) and (c) are replotted against time in Supplemental Fig. 1 to better illustrate response kinetics.

L*β*T2 cells were stimulated continuously with varied concentrations of GnRH for 5, 15, 30, 60, 120, or 240 minutes before staining and imaging. Image analysis revealed that GnRH caused the expected rapid (maximal or near maximal at 5 minutes) and concentration-dependent increases in ppERK [Fig. 1(a)] [9, 17, 24]. The population-averaged data shown are derived from >10^6^ cells, and representative frequency distribution plots are shown in Supplemental Fig. 1C. The single-cell data for each of the GnRH concentration–response curves was used to calculate MI between GnRH and ppERK [I(ppERK;GnRH)], and this value increased rapidly to ~0.7 bit at 5 minutes with a gradual reduction to ~0.5 bit at 240 minutes [Fig. 1(b)]. Similar experiments were undertaken with L*β*T2 cells transduced with Ad NFAT-EFP before staining, imaging, and calculation of NFAT-NF. Again, GnRH caused a concentration-dependent increase in NFAT-NF [Fig. 1(c)] that was slower than the effect on ppERK (maximal or near maximal at 60 minutes). Frequency distribution plots are shown in Supplemental Fig. 1D, and the single-cell measures were used to calculate MI between GnRH and NFAT-NF. As shown Fig. 1(d), information transfer to NFAT was lower than to ERK as I(NFAT-NF;GnRH) values were lower, increasing to a maximum (~0.3 bit) at 60 minutes with a gradual reduction toward 240 minutes. For clarity, the data in Fig. 1(a) and 1(c) are replotted against time in Supplemental Fig. 1, and this reveals that the population-averaged ppERK response to GnRH was more sustained with the higher concentrations of GnRH than with 10^−11^ to 10^−9^ M GnRH. This reflects the time-dependent rightward shift in the GnRH concentration–response curves evident in Fig. 1(a) [*i.e.*, the 50% effective concentration (EC_50_) for GnRH was ∼1 nM at 5 minutes and 46 nM at 240 minutes; see the Fig. 1 legend for all EC_50_ values). In this study, it is important to recognize that all of the individual cell measures underlying the full concentration–response curves were used for the MI calculations, and that these MI values would not be expected to be influenced by the time-dependent reduction in GnRH potency (as measured by EC_50_ values) so long as the GnRH concentrations used encompass the full dynamic range of the response. Accordingly, these data collectively reveal that MI can be used to measure information transfer via endogenous GnRHR to ERK and NFAT, and that for each output and time point considered, the MI values approximately paralleled the dynamic range observed for the population-averaged responses.

### B. Joint Sensing of ERK and NFAT Signaling

**Figure 2.**
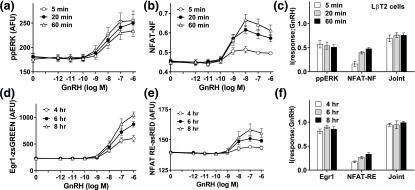
Joint sensing of ERK and NFAT signaling in L*β*T2 cells. For (a)–(c), L*β*T2 cells transduced with Ad NFAT-EFP were continuously stimulated 5, 20, or 60 minutes with 0 or 10^−12^ to 10^−6^ M GnRH as indicated before being fixed, stained (DAPI and ppERK), and imaged for ppERK and NFAT-NF as described for Fig.1, except that in this case both were measured in identical cells. The single-cell data from the full concentration response curves were then used to calculate MI between GnRH concentration and each response (ppERK or NFAT-NF) and also the joint MI between GnRH and both responses (Joint). For (d) and (e), L*β*T2 cells transduced with Ad Egr1-zsGREEN and Ad NFAT RE-asRED were stimulated for 4, 6, or 8 hours with 0 or 10^−12^ to 10^−6^ M GnRH as indicated before being fixed, stained (DAPI), and imaged to quantify zsGREEN and asRED. The single-cell data were then used to calculate MI between GnRH and the expression level for each reporter and also the joint MI between GnRH and both reporters (Joint). The data shown are means ± standard error of the mean (n = 5 to n = 7) for population-averaged measures of ppERK (a), NFAT translocation (NFAT-NF, b), Egr1-driven zsGREEN expression (d), and NFAT RE-driven asRED expression (e), as well as I(response;GnRH) (c and f) in bits. Log EC_50_ values for (a) were −8.24 ± 0.07, −8.01 ± 0.08, and −7.76 ± 0.16 (n = 7) at 5, 20, and 60 minutes, respectively, and one-way analysis of variance revealed that time was not a significant source of variation (*P* > 0.05).

In the previous experiments, MI values were always <1 bit despite system inputs of 3 bits (*i.e.*, 2^3^ different GnRH concentrations). This implies that most information from the environment is lost through signaling, but an important alternative possibility is that sensing of multiple pathways within the network actually mitigates any such loss. We addressed this by measuring ERK phosphorylation and NFAT translocation responses in the same population of Ad NFAT-EFP–transduced L*β*T2 cells. As shown (Fig. 2), GnRH caused the expected concentration-dependent and time-dependent increases in ppERK and NFAT-NF with very similar I(ppERK;GnRH) values (~0.5 bit) at 5, 20, and 60 minutes, whereas I(NFAT-NF;GnRH) values increased from 5 to 60 minutes. Joint MI values were comparable at all time points and were always greater than MI values for either response alone, but the increase was modest (maximally from ~0.5 to ~0.7 bit), so the additional information gained by joint sensing was small. Similar experiments were performed with cells transduced with Ad Egr1-zsGREEN and Ad NFAT RE-asRED (as imaging readouts for ERK-driven and NFAT-driven transcription, respectively) and stimulated for 4, 6, or 8 hours before imaging. Again, GnRH caused concentration- and time-dependent increases in expression of both reporters, although as expected these responses were much slower (note the different time points used for the upper and lower panels in Fig. 2). MI values were ~0.8 bit at all three time points for the Egr1 reporter and were considerably lower (0.2 to 0.3 bit) for the NFAT-RE reporter. Joint MI values were greater than for either reporter alone but the increase was modest (from ~0.8 to ~0.9 bit), so although additional information is gained by sensing both effectors, this increase was again small. Similar experiments were undertaken with HeLa cells, which yielded similar conclusions (Supplemental Fig. 2). Joint MI values were greater than for I(ppERK;GnRH) and I(NFAT-NF;GnRH) but the increase was small (*i.e.*, from ~0.7 to ~0.8 bit for 5-minute responses), and although joint MI values were greater than for I(Egr1-zsGREEN;GnRH) or I(NFAT-RE-asRED;GnRH), the increase was again small (*i.e.*, from ~0.35 to ~0.45 bit for 8-hour responses). Interestingly, these experiments also revealed signal bias for information transfer in these two models. GnRH tended to cause more sustained increases in ppERK in L*β*T2 cells than in Ad GnRHR-transduced HeLa cells [compare Fig. 2(a) and Supplemental Fig. 2A, particularly at the maximally effective concentrations]. Because transcriptional effects of ERK are most pronounced with sustained stimulation [32], it is not surprising that GnRH had a more pronounced effect on Egr1-zsGREEN expression in L*β*T2 cells than it did in HeLa cells [compare Fig. 2(d) with Supplemental Fig. 2D]. There was also more information transfer in L*β*T2 cells, as I(Egr1-zsGREEN;GnRH) was ~0.8 bit in L*β*T2 cells and only ~0.2 bit in HeLa cells [compare Fig. 2(f) and Supplemental Fig. 2F]. In contrast, for the NFAT-driven transcriptional response as I(NFAT-RE-asRED), values were lower in L*β*T2 cells than in HeLa cells [compare panels (e) and (f) in Fig. 2 and Supplemental Fig. 2]. Taken together, these data reveal that in Ad GnRHR–transduced HeLa cells, more information about GnRH concentration is transferred to the transcriptome via NFAT than via ERK, but that the opposite is true in L*β*T2 cells. Moreover, for both cell types information transfer is increased by joint sensing of NFAT and ERK, although this additional information is very small (always <50%).

### C. Sensing Response Trajectories

**Figure 3.**
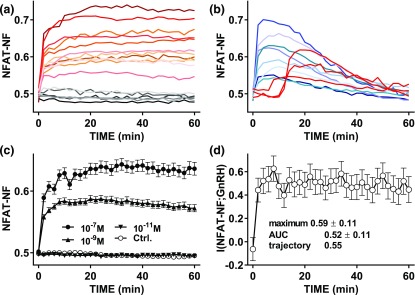
Sensing dynamics and live cell NFAT-EFP imaging. HeLa cells transduced with Ad GnRHR and Ad NFAT-EFP were stained with Hoechst dye (for imaging of nuclei) transferred to live cell imaging medium and imaged at 37°C both before and during continuous stimulation with 0, 10^−11^, 10^−9^, or 10^−7^ M GnRH. Automated image analysis algorithms were used to calculate the nuclear fraction of NFAT-EFP (NFAT-NF, calculated for each cell and at each time-point), and individual cells were tracked over time. The individual cell time courses were then inspected for removal of cells in which tracking had failed or had time 0 NFAT-NF values of <0.4 or >0.55 (this removed <10% of cells as outliers). In (a) and (b), responses are shown of representative individual cells stimulated with 10^−9^ M GnRH and selected to illustrate distinct response patterns, including little or no response [gray shade traces in (a)], rapid and sustained increases [red shade traces in (a)], rapid but not sustained [blue shade traces in (b)], and delayed [red traces in (b)] responses. Most cells (>50% to 75%) showed rapid and sustained responses and very few showed delayed responses (3 of 166 for this data set). Population-averaged responses for all tracked cells are shown in (c) (mean ± standard error of the mean, n = 72 to n = 167). I(NFAT-NF;GnRH) values for the tracked cells were calculated for each individual time point and are shown in (d) (mean ± standard deviation). I(NFAT-NF;GnRH) values were also calculated from the same tracked cells using the AUC during the full 60 minutes as the response, and also using three time points to taking individual cell response trajectories into account as described in *Materials and Methods*. These values are also shown (along with the maximum snapshot value) in (d).

The previous data were obtained by imaging fixed cells, and such snapshot data may well underestimate the information available to cells sensing response trajectories over time. We addressed this for the Ca^2+^/calmodulin/calcineurin/NFAT pathway by live cell imaging of Ad NFAT-EFP– and Ad GnRHR–transduced HeLa cells and cell tracking. As shown (Fig. 3), the responses of individual cells to GnRH were highly variable, with some cells showing rapid and sustained increases in NFAT-NF [red shade traces in Fig. 3(a)], whereas some showed little or no response [gray shade traces in Fig. 3(a)] and others showed rapid and transient responses [blue shade traces in Fig. 3(b)] or delayed responses [red traces in Fig. 3(b)]. The rapid and sustained responses were most prevalent (>50% to 75%), whereas very few cells showed delayed responses (3 of 166 for this data set). The population-averaged responses increased to maxima at 15 to 60 minutes [Fig. 3(c)], and MI between GnRH and NFAT-NF was ~0.5 bit at all time points measured. These data demonstrate that we have not underestimated I(NFAT-NF;GnRH) by missing a specific time point, and they are broadly consistent with the snapshot data shown (for 5, 20, and 60 minutes) in Supplemental Fig. 2. Using the live cell data we could also calculate I(NFAT-NF;GnRH) using the area under the curve (AUC) for the tracked cell responses [I(NFAT-NF AUC;GnRH)] or using three time points [I(NFAT-NF trajectory;GnRH)], and these values were ~0.52 and ~0.55 bit, respectively (as compared with an average of 0.48 bit for the snapshot data). Accordingly, although sensing of response trajectory can theoretically increase the MI values, sensing over time provided little or no increase in information transfer via GnRHR to NFAT.

### D. Sensing GnRH Pulses

**Figure 4.**
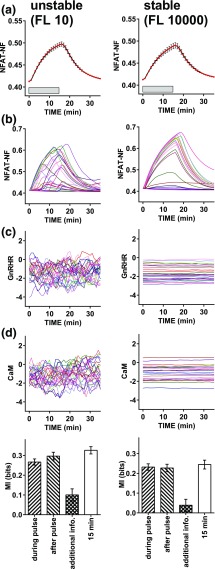
Mixed mechanistic and probabilistic modeling of NFAT responses to GnRH pulses: sensing during and beyond the GnRH pulse. We adapted a deterministic model of GnRH signaling [31] and used it to simulate responses during and immediately after a 15-minute pulse of 0, 10^−11^, 10^−9^, and 10^−7^ M GnRH. We introduced heterogeneity into the concentration of two effectors, the GnRHR and calmodulin (CaM, which equates to M in Ref. [31]). For each of these we added synthesis and degradation to the model and introduced cell–cell variability by allowing them to fluctuate, simulating unstable effector expression with an FL of 10 and stable effector expression with an FL of 10,000. (a) Population-averaged data for simulations with 10^−7^ M GnRH with unstable (left panels) or stable (right panels) effector expression (means ± standard error of the mean, n = 1000). (b) Representative traces for 25 individual cells. The GnRHR and CaM concentrations for the same representative cells are shown in (c) and (d). The individual cell simulation data were used to calculate MI using (as response) either the 15-minute snapshot data or the AUCs during the 15-minute GnRH pulse or the following 15 minutes. The bar charts show these MI values (means ± standard deviation) for the unstable and stable scenarios (left and right panels, respectively) along with the additional information gained by sensing both during and beyond the pulse, calculated as described in *Materials and Methods*. Note that information transfer during and beyond the pulse are comparable to one another (and to the snapshot values) and that there is little information gained by sensing both, particularly when effector expression is stable.

Physiologically GnRH is secreted in pulses, and signaling can continue beyond the GnRH pulse [33], raising the question of how much information is gained by sensing during and after the pulse. We initially addressed this theoretically by developing a hybrid (deterministic/stochastic) model for GnRH signaling to NFAT. To do so we simplified a deterministic model of GnRH signaling that was adapted from an earlier version [31] by removal of the ERK signaling pathway and transcription regulation steps, and by altering the NFAT translocation parameters to better fit the response dynamics shown for wet laboratory data in Fig.6. This was used to simulate responses during and immediately after a 15-minute pulse of 0, 10^−11^, 10^−9^, and 10^−7^ M GnRH. We introduced stochasticity into the concentration of two effectors: GnRHR and calmodulin. We allowed each of these to fluctuate, considering both unstable effector expression with an FL of 10 minutes and stable effector expression with an FL of 10,000 minutes. We ran simulations for 1000 cells at each combination of GnRH concentration and FL, so that MI values could be calculated. As expected (Fig. 4), population-averaged simulation data revealed NFAT-NF responses for the stable and unstable systems that had comparable means and variance [Fig. 4(a)] despite the fact that individual cell response trajectories were more variable [Fig. 4(b)] with the more unstable effector concentrations [Fig. 4(c) and 4(d)]. The individual cell simulation data were used to calculate MI values using (as response) the AUCs of the individual cell traces either during the 15-minute GnRH pulse or in the following 15 minutes. These values were comparable (*i.e.*, 0.27 ± 0.02 and 0.30 ± 0.02 bit during and after the GnRH pulse with FL of 10). As in live cell data previously shown, MI values calculated using the snapshot data were comparable to those calculated using response AUCs (Fig. 4). Moreover, when we calculated the additional information gained by sensing both during and after the GnRH pulse (Fig. 4, cross-hatched bars), this was low, but it was greater for the unstable scenario (0.10 ± 0.03 with FL of 10 and 0.03 ± 0.03 with FL of 10,000). Thus, these simulations suggest that for NFAT signaling, the cells can gain as much information about GnRH concentration after the pulse as they do within it, and that the additional information to be gained by sensing both would be greatest for conditions in which effector concentrations fluctuate rapidly.

**Figure 5.**
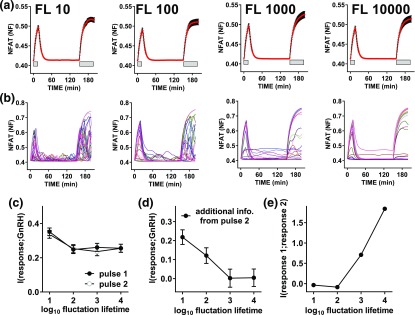
Mixed mechanistic and probabilistic modeling of NFAT responses to GnRH pulses: sensing two pulses. The data in this figure are from a larger series of simulations (from those in Fig.4) in which we considered four effector stabilities, and also followed the first 15-minute GnRH pulse with a 120-minute interval and then a second pulse (30 minutes, at the same GnRH concentration as the first) so that we could calculate not only information transfer during each pulse, but also the additional information gained by sensing both. Again, we ran simulations for 1000 cells at each GnRH concentration/effector stability combination. (a) Population-averaged data for simulations with 10^−7^ M GnRH at each of four stabilities (FL of 10, 100, 1000, and 10,000, means ± standard error of the mean, n = 1000). (b) Representative traces for 25 individual cells. (c) MI between GnRH and the predicted NFAT-NF response using AUCs for pulse 1 or pulse 2. (d) Additional information gained by sensing both pulses. (e) MI between responses in pulses 1 and 2. In (c)–(e), results are plotted against FL (where log_10_ FL values of 1 and 4 represent the most unstable and stable scenario, respectively) and show means ± standard deviation. Note that at any given time point, mean values and variance are comparable for NFAT-NF (a), as well as for GnRHR and CaM (not shown), but as effector stability is increased this increases MI between the pulse 1 and pulse 2 responses (e) and reduces additional information gained from the 2-second pulse (d).

The data described previously are from a larger series of simulations in which we also considered the question of how much additional information can be gained by sensing a second GnRH pulse. Thus the full simulations included a 15-minute pulse of GnRH followed by an interval of 135 minutes and then a second (60-minute) pulse of GnRH at the same concentration as the first pulse. These simulations were run with four effector stabilities (FL of 10, 100, 1000, and 10,000 minutes) and, again, population-averaged NFAT-NF responses for the stable and unstable systems had comparable means and variance [Fig. 5(a)] despite that individual cell response trajectories were more variable with the more unstable effectors [compare left and right panels in Fig. 5(b)]. When effector stability is high the cells showing greatest responses in pulse 1 also show large responses in pulse 2, whereas this is not the case when effector stability is low [compare left and right panels in Fig. 5(b)]. I(NFAT-NF;GnRH) values calculated using AUCs (for the first 15 minutes of stimulation in either pulse) were ~0.25 to 0.4 bit and were comparable for pulse 1 and pulse 2 irrespective of effector stability [Fig. 5(c)]. Additional information gained by sensing both pulses was negligible with high effector stability but increased to >0.2 bit at the lowest effector stability [Fig. 5(c)]. We also calculated the MI between the pulse 1 and pulse 2 responses and this increased from 0 to ~1.8 bits as FL was increased from 10 to 10,000 minutes (*i.e.*, as effector stability increased). Thus, these simulations reveal that the additional information from the second pulse is dependent on the nature of the variation. If the heterogeneity reflected a broad distribution of effector concentrations that was constant over time, then the response in pulse 2 would be predictive of that in pulse 1 and there would be no additional information from sensing both. This is the situation approached at FL of 10,000 minutes where additional information from the second pulse is negligible and the MI between pulse 1 and 2 responses is high. In contrast, if the source of the heterogeneity were random or changed rapidly over time, the response in pulse 2 would be less predictive of that in pulse 1, so additional information would be obtained by sensing both. This is the scenario with FL of 10, where additional information from the second pulse is relatively high [Fig. 5(d)] and the MI between pulse 1 and 2 responses is low [Fig. 5(e)].

**Figure 6.**
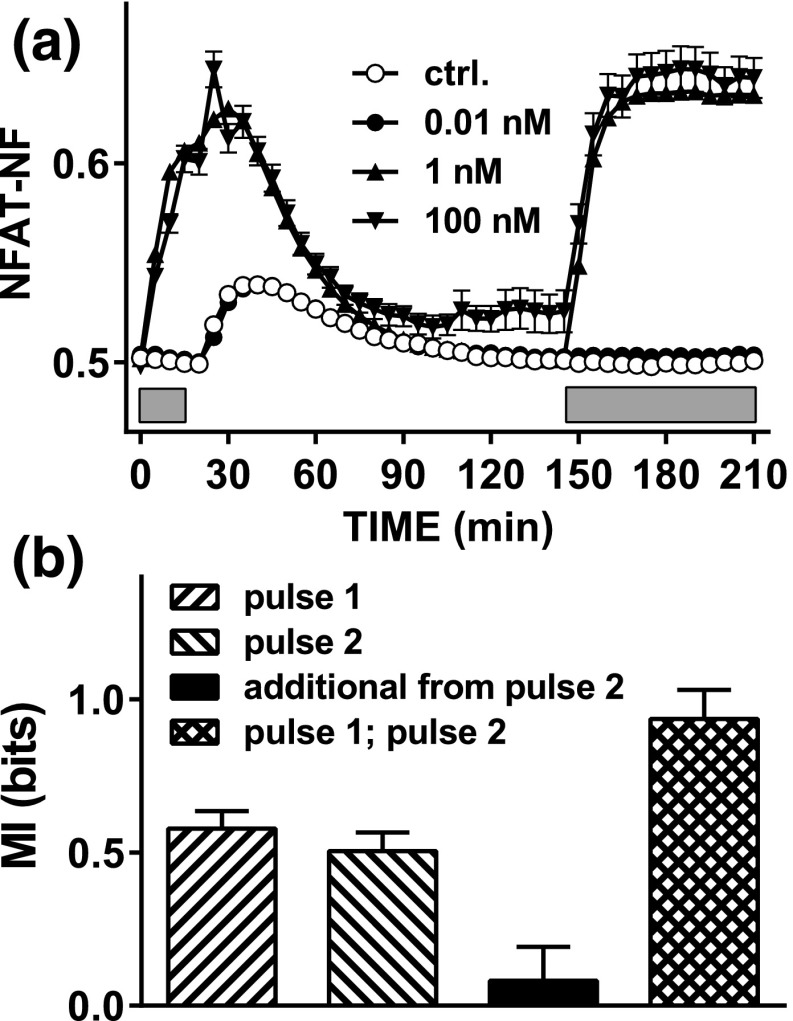
Sensing dynamics with repeated stimulation and live cell NFAT-EFP imaging. (a) HeLa cells transduced with Ad GnRHR and Ad NFAT-EFP were stained with Hoechst dye transferred to live cell imaging medium and imaged at 37°C both before and during stimulation with 0, 10^−11^, 10^−9^, or 10^−7^ M GnRH for 15 minutes (first gray bar). The plate was then removed from the stage, cells were washed (with PBS, three times during 5 minutes), and the plate was returned to the stage. The cells were imaged for a further 2 hours before repeat stimulation (second gray bar) for 60 minutes using the same concentrations of GnRH. Image analysis and data processing were as described under Fig. 4. The data shown are pooled from the tracked cells in three repeated experiments (mean ± standard error of the mean, n = 67 to n = 489). (b) MI values calculated using the AUC for the first 15 minutes of stimulation with GnRH in pulse 1 or pulse 2, the additional information gained by sensing both, and the MI between responses in pulse 1 and pulse 2. Note that these data are consistent with the stable effector scenario of Fig. 5, with little additional information gained by sensing both pulses because responses in one pulse are highly predictive of those in the other.

The previous simulations illustrate conditions where additional information is, or is not, gained by sensing during and after one pulse, or by sensing two consecutive pulses, raising the question of what actually happens in GnRH-stimulated cells. To test this, we stimulated Ad GnRHR– and Ad NFAT-EFP–transduced HeLa cells with two separate pulses of GnRH at 0, 10^−11^, 10^−9^, and 10^−7^ M followed by imaging and individual cell tracking as outlined earlier. The first pulse was for 15 minutes and was terminated by washing (four times) to remove the stimulus, followed by an interval of 135 minutes and then stimulation (60 minutes) with GnRH at the same concentration as had been used for the first pulse (Fig. 6). I(NFAT-NF;GnRH) values calculated using the AUCs for the individual cell response during the first 15 minutes of each pulse were comparable to one another (0.58 ± 0.06 and 0.50 ± 0.06 for pulses 1 and 2, respectively) and the values obtained in the single-pulse experiment (Fig. 3). An unexpected observation in this study was that the wash itself caused a small and transient increase in NFAT-NF (open circles in Fig. 6). This likely reflects the effect of mechanical stimulation on cytoplasmic Ca^2+^ concentration and prevents meaningful comparison of information transfer during and beyond the first GnRH pulse. Nevertheless, we were able to calculate the additional information due to sensing both pulses (~0.1 bit) and the MI between responses in pulses 2 and 1 (~1.0 bit). Accordingly, the wet laboratory data parallel the situation simulated in Fig. 5 with high effector stability, implying that the sources of cell–cell heterogeneity are relatively stable over time so that there is little additional information to be gained from sensing the second pulse, at least in this time frame and under our experimental conditions.

## 3. Discussion

Information theory–derived statistical measures, such as the MI between a stimulus and a response, can be used to quantify information transfer via cell signaling pathways, taking into account both the dynamic range and the cell–cell variability of responses. Importantly, MI can be measured without knowledge of the transduction mechanism, and MI values are unaffected by nonlinear transformations of the signal or response so they are not influenced by nonlinear input–output relationships prevalent in cell signaling pathways [1]. In this study, we have used this approach to estimate information transfer via GnRHR to ERK and to NFAT, placing emphasis on the relevance of dynamics for GnRH sensing. For each effector we found that most of the information available from the environment was lost through signaling (*i.e.*, that with 3 bits of information available, <1 bit was transferred) and that although information transfer could be increased by joint sensing (of both effectors) and by trajectory sensing, the additional information we observed was small. We also developed a hybrid mechanist/probabilistic model of GnRH signaling to NFAT and used this to simulate responses to constant or pulsatile GnRH. The model predicts that a second pulse of GnRH will provide considerable additional information when cell–cell variability reflects rapidly changing effector levels, but not when it is due to variation in effectors, that is stable over the time frame of the repeated pulses. Live cell imaging experiments closely paralleled the latter scenario, yielding very little additional information from a second GnRH pulse. This study addresses the information gain from repeat hormone stimulation, and in general it reveals that the additional information available from sensing repeat pulses depends on stability of network componentry.

The work described in the present study extends a recent study in which we used the same information theoretic approach to quantify information transfer via GnRHR to ERK in HeLa cells transduced for expression of GnRHR [9]. This revealed MI values [I(ppERK;GnRH)] of <1 bit, which implies that the individual cells cannot unambiguously distinguish between even two states of the environment (*i.e.*,two GnRH concentrations). This is consistent with information transfer estimates for other receptors and/or effectors [2–4, 7] but is still somewhat surprising, as it is well known that GnRH elicits graded signaling, gene expression, and gonadotropin secretion over broad concentration ranges in many models [13, 14, 20–23]. In the previous study we considered the possibility that I(ppERK;GnRH) values of <1 bit might reflect negative feedback, as it is well established that ERK responses are shaped by multiple feedback pathways [32, 34–36]. We found that ERK-mediated negative feedback could reduce information transfer by reducing response amplitude, but it could also increase it by reducing cell–cell variability. Thus, information transfer was maximal with intermediate feedback but, nevertheless, was always <1 bit. Another possible explanation for these low MI values is that little information is transferred via GnRHR to ERK because with this bifurcating signaling system, most information actually passes via the PLC/Ca^2+^/calmodulin pathway. Alternatively, information transfer could have been underestimated by use of a heterologous receptor expression system, or by simply missing the optimal time point for its measurement. However, the data in the present study argue against each of these possibilities. Notably, the fixed cell experiments (Figs. 1 and 2; Supplemental Figs. 1 and 2) reveal that information transfer to NFAT is actually lower than to ERK at most time points, that information transfer is comparable for heterologously expressed GnRHR in HeLa cells and for endogenous GnRHR of L*β*T2 cells, and that for all experimental readouts and both models, MI values were <1 at all time points considered. Another possibility is that GnRH sensing is underestimated by consideration of a single pathway or effector because simultaneous activation of multiple effectors improves it. To address this, we imaged ERK and NFAT responses in identical cells to calculate joint MI (Fig. 2; Supplemental Fig. 2). This revealed that for both cellular models and for both upstream activation readouts (ppERK and NFAT-NF measures) and downstream transcriptional readouts (Egr1-driven zsGREEN and NFAT-RE–driven asRED expression) joint MI values were always greater than for either measure alone. However, the increase was relatively small (often <0.1 bit). Thus, the present study confirms the previous observation that most information about GnRH concentration is lost through signaling and, importantly, extends it by showing that I(response;GnRH) values are <1 bit over full time courses with endogenous GnRHR and for each of the responses considered, alone and in combination.

A particularly interesting observation in this study is that I(ppERK;GnRH), I(NFAT-NF;GnRH), and joint MI values showed different time dependencies. This was most obvious in HeLa cells (Supplemental Fig. 2C) where I(ppERK;GnRH) dropped from 0.64 to 0.09 bit from 5 to 60 minutes whereas I(NFAT-NF;GnRH) remained unaltered (at ~0.45 bit), and joint MI showed only a small reduction (from 0.73 to 0.53 bit) during the same period. Recent work on growth factor–stimulated signaling [7] revealed how concomitant activation of distinct pathways made information transfer robust to pharmacological manipulation because compensation occurred (*i.e.*, where one pathway was inhibited but information transfer through another was retained). Our data reveal a related situation where concomitant activation of two pathways makes information transfer robust to the reduction in sensing due to adaptation in one of them over time. In this study, it is important to recognize that this robustness, in terms of information transfer, does not equate to biological redundancy. Consider the situation where an extracellular stimulus elicits single-cell ERK and NFAT responses that are perfectly correlated with one another, yet ERK and NFAT mediate different responses by activation of different effectors. In this scenario, ablation of ERK would not reduce the information the cell has about hormone concentration in its environment but would abrogate the ERK-mediated response. Similarly, ablation of NFAT would not reduce the information the cell has about hormone concentration in its environment but would abrogate the NFAT-mediated response. Thus, in this bifurcating signaling system, the observed robustness in information transfer from receptor to ERK and NFAT tells us nothing about information transfer downstream of ERK and NFAT.

Taken together, the data outlined earlier reveal that GnRH-responsive cells gain more information by sensing both ERK and NFAT pathways, but that this additional information is rather modest, and the concomitant activation of both pathways may serve instead to ensure robust information transfer. However, they still do not explain the relatively low I(response;GnRH) values obtained, so we also considered the possibility that single time point measures greatly underestimate information transfer. Indeed, the time courses in Fig. 2 reveal I(ppERK;GnRH) and I (NFAT-NF;GnRH) values at 60 minutes to be higher than those at 240 minutes, but this clearly does not mean that the cells had obtained less information during 240 minutes than they had during 60 minutes. Instead, it shows that the 240-minute snapshot underestimates the amount of information transferred. We recently argued on the basis of transcriptional readouts for GnRHR signaling to ERK that cells must gain additional information by sensing response trajectory [9], and in the present study we have taken a more direct approach, using live cell imaging of NFAT1c-EFP translocation responses and cell tracking. This enabled us to calculate MI values not only using snapshots of individual cell responses at specific time points, but also using single cell–integrated responses or response trajectories. In the first instance we simply stimulated GnRHR-expressing HeLa cells for 60 minutes with GnRH (0 or 10^−11^ to 10^−9^ M) and, consistent with the fixed-cell experiments, this revealed I(NFAT-NF;GnRH) values of ~0.5 bit at all time points (Fig. 4). MI values were higher for the integrated readout and when trajectory was considered but the increase was small (<0.1 bit), so little or no additional information was gained by sensing the NFAT translocation response trajectory. However, GnRH is secreted in pulses, so we were particularly interested in this scenario and explored it theoretically by developing a hybrid (deterministic/stochastic) model of the GnRH signaling pathway (from GnRHR to NFAT). We introduced heterogeneity in the expression levels of the receptor and calmodulin, and simulations revealed the potential for additional information to be gained by sensing beyond the GnRH pulse (Fig. 5), as well as by sensing a second GnRH pulse (Fig. 6). Because cell–cell variability could reflect stable differences in system componentry or, alternatively, differences that are either random or rapidly changing over time, we considered both possibilities by varying the timescale at which the total amount of GnRHR and calmodulin fluctuate. Model simulations suggested that sensing the second GnRH pulse would provide little or no additional information if the effectors were stable (such that responses in one pulse were predictive of responses in the other), and the data from dual-pulse live cell imaging experiments were consistent with this scenario. However, because the additional information is related to the reliability with which responses in one pulse predict those in the other, we would anticipate that the information gained from a second pulse would be increased by increasing the time between the pulses. More generally, if we consider two brief pulses of stimulus and a system where cell–cell variability reflects the concentration of effectors, the additional information gained from the second pulse will increase as the interpulse interval increases and will decrease as effector stability increases.

The data described also relate to the more general question of why pulsatile signals are so prevalent in biological systems. We have recently explored this by deterministic modeling of pulsatile stimulation with varied pulse width, amplitude, and frequency [31]. This revealed how pulsatility can increase signaling efficiency in the sense that with pulsatile and constant stimuli and identical input integrals, the system output can be much greater with the pulsatile stimulation. This occurs largely because signaling continues in the intervals between the pulse, and the extent of this depends on activation and inactivation rates that will differ for different effectors. Consequently, with pulsatile stimuli, input–output relationships for different effectors are not superimposable, and this can give output-specific frequency–response relationships where no such specificity occurs with concentration–response relationships [31]. In this study, we considered a third possibility, that with pulsatility there is a substantial information gain from repeated stimuli; however, our data argued against this, at least for GnRH stimulation in the experimental paradigms considered. Another important general observation in this study is that even when joint sensing or trajectory are considered, our I(response;GnRH) values were always <1. Thus, the individual GnRH-responsive cells cannot distinguish between even two GnRH concentrations, and this contrasts to numerous published studies showing dose-dependent effects of GnRH on populations of GnRH-responsive cells (including the examples in this study). Clearly cell populations can discern GnRH concentrations more effectively than individual cells, and this could reflect averaging of responses over multiple cells and/or cell–cell communication providing additional information. The latter possibility is of particular interest, as gonadotropes communicate with one another via gap junctions [37, 38], and such communication could improve sensing. Thus, although it is individual cells that have to sense and respond to GnRH in their environment, these decisions could well be informed by additional information from their neighbors.

In summary, we have used single-cell measures to quantify GnRHR-mediated information transfer to ERK and NFAT and found that signaling is inefficient, in the sense that most information about GnRH concentration in the environment is lost through signaling. Information transfer was increased by joint sensing of both pathways, but the additional information was small, and little or no additional information was gained by sensing individual cell NFAT response trajectories over time. Because GnRH secretion is pulsatile, we also explored the sensing of input dynamics by developing a model of GnRH signaling that suggests, for NFAT signaling at least, that cells gain as much information by sensing beyond the GnRH pulse as they do during it. These simulations also highlight the importance of effector stability because when cell–cell variability reflects differences in rapidly fluctuating effector levels, additional information is predicted to be gained by sensing two GnRH pulses. In contrast, where there is comparable cell–cell variability in slowly fluctuating effector levels, responses in one pulse are predictive of those in another, so there is little additional information to be gained by sensing both. Parallel wet laboratory experiments suggest that the latter scenario holds true for GnRH signaling via the PLC/ Ca^2+^/calmodulin/calcineurin/NFAT pathway, and that (within the time frames considered) cell–cell variability is low, so that response trajectories are reproducible from pulse to pulse. Thus, although the pulsatile input can increase signaling efficiency and specificity [31], these first information theoretic studies of dynamic GnRH sensing suggest that the amount of information the cell has about GnRH concentration in its environment is not greatly increased by sensing additional pulses.
